# Engineering of sugar transporters for improvement of xylose utilization during high-temperature alcoholic fermentation in *Ogataea polymorpha* yeast

**DOI:** 10.1186/s12934-020-01354-9

**Published:** 2020-04-25

**Authors:** Roksolana Vasylyshyn, Olena Kurylenko, Justyna Ruchala, Nadiya Shevchuk, Neringa Kuliesiene, Galina Khroustalyova, Alexander Rapoport, Rimantas Daugelavicius, Kostyantyn Dmytruk, Andriy Sibirny

**Affiliations:** 1 Department of Molecular Genetics and Biotechnology, Institute of Cell Biology, NAS of Ukraine, Drahomanov Street, 14/16, Lviv, 79005 Ukraine; 2 Department of Microbiology and Biotechnology, University of Rzeszow, Zelwerowicza 4, Rzeszow, 35-601 Poland; 3 Department of Biochemistry, Faculty of Natural Sciences, Vytautas Magnus University, Vileikos 8, 44404 Kaunas, Lithuania; 4 Laboratory of Cell Biology, Institute of Microbiology and Biotechnology, University of Latvia, Jelgavas Str., 1-537, Riga, 1004 Latvia; 5 Latvian - Ukrainian Joint International Laboratory of Microbial Cell Biology, Jelgavas Str., 1, Riga LV-1004, Latvia, Drahomanov Street, 14/16, Lviv, 79005 Ukraine

**Keywords:** Xylose, *Ogataea* (*Hansenula*) *polymorpha*, Xylose transporters, High-temperature alcoholic fermentation

## Abstract

**Background:**

Xylose transport is one of the bottlenecks in the conversion of lignocellulosic biomass to ethanol. Xylose consumption by the wild-type strains of xylose-utilizing yeasts occurs once glucose is depleted resulting in a long fermentation process and overall slow and incomplete conversion of sugars liberated from lignocellulosic hydrolysates. Therefore, the engineering of endogenous transporters for the facilitation of glucose-xylose co-consumption is an important prerequisite for efficient ethanol production from lignocellulosic hydrolysates.

**Results:**

In this study, several engineering approaches formerly used for the low-affinity glucose transporters in *Saccharomyces cerevisiae*, were successfully applied for earlier identified transporter Hxt1 in *Ogataea polymorpha* to improve xylose consumption (engineering involved asparagine substitution to alanine at position 358 and replacement of N-terminal lysine residues predicted to be the target of ubiquitination for arginine residues). Moreover, the modified versions of *S. cerevisiae* Hxt7 and Gal2 transporters also led to improved xylose fermentation when expressed in *O. polymorpha*.

**Conclusions:**

The *O. polymorpha* strains with modified Hxt1 were characterized by simultaneous utilization of both glucose and xylose, in contrast to the wild-type and parental strain with elevated ethanol production from xylose. When the engineered Hxt1 transporter was introduced into constructed earlier advanced ethanol producer form xylose, the resulting strain showed further increase in ethanol accumulation during xylose fermentation. The overexpression of heterologous *S. cerevisiae* Gal2 had a less profound positive effects on sugars uptake rate, while overexpression of Hxt7 revealed the least impact on sugars consumption.

## Background

The thermotolerant methylotrophic yeast *Ogataea* (*Hansenula*) *polymorpha* belongs to the native xylose-fermenting yeast species with the ability to grow and ferment xylose and other sugars of lignocellulose at elevated temperatures (up to 50 °C). Such property of this yeast defines it as a good candidate for the development of an efficient process for simultaneous saccharification and fermentation (SSF) of lignocellulosic hydrolyzates [1]. However, to be economically viable, the main characteristics of xylose fermentation in *O. polymorpha,* like ethanol yield, productivity, and co-fermentation with glucose, have to be improved.

The Advanced *O. polymorpha* ethanol producers from xylose were obtained by a combination of the methods of metabolic engineering and classical selection [2, 3]. Although such recombinant strains were characterized by 25-30-fold improved ethanol production from xylose as compared to the wild-type strain, xylose uptake was slow and incomplete. It is known that for xylose-utilizing yeasts, glucose appeared to be the preferred sugar, being consumed first during mixed sugar fermentation [4–6]. One of the main drawbacks of the efficient conversion of sugars from the lignocellulosic hydrolysates could be an insufficient transport of xylose which additionally is competitively inhibited by glucose. One may assume that improvement of xylose utilization in *O. polymorpha*, similarly to *Saccharomyces cerevisiae*, could be partially achieved by enhancement of the efficiency of transporters mediating the uptake of xylose. This could be achieved by expression of the specific xylose transporters from other organisms in *O. polymorpha* and/or by the identification and engineering the native transporters with improved ability to transport xylose [7].

*Saccharomyces cerevisiae* lacks a xylose-specific transport system but contains hexose transporters (Hxt) that mediate glucose uptake. Several Hxt proteins also mediate uptake of xylose albeit with lower affinity. A multiple deletion strain lacking the key transporters Hxt1-17 and Gal2 was found to be unable to grow on xylose and glucose. In this strain, the growth on xylose could be restored by the reintroduction of Hxt1, Hxt2, Hxt4, Hxt5, Hxt7, or Gal2 [8]. Different strategies were applied for the engineering of endogenous hexose transporters to construct glucose-insensitive xylose transporters by modification of crucial residues involved in glucose affinity, selectivity, and substrate translocation [7, 9, 10]. Many native pentose-assimilating species have low-affinity and high-affinity sugar transport systems for xylose uptake, both, diffusion facilitating transporters and proton symporters. More than 80 heterologous sugar transporters have already been expressed in *S. cerevisiae*. Among them, Sut1, Sut2, Xut1, Xut3 (Xyp33), Xut4, Xyp29 (STL12), Sut3 (Xyp37) from *Scheffersomyces stipitis*, Gxs1 and Gxf1 from *Candida intermedia*, At5g59250 from *Arabidopsis thaliana*, An29-2 and An25 from *Neurospora crassa*, xtrD from *Aspergillus nidulans*, MgT05196 from *Meyerozyma guilliermondii* and Xylh from *Debaryomyces hansenii* restored the ability of *S. cerevisiae* HXT null-mutant to transport xylose. Three of them were characterized to be the xylose-specific transporters: An25 and Xyp29 are xylose facilitators, with no glucose uptake activity, and Xut1 is able to transport glucose, but with lower affinity. However, the native hexose transporters showed the highest xylose uptake activities, even when compared with xylose-specific transporters, like CiGsx1 (F38I39M40), SsXyp29, NcAn25, and SsXut1 [11, 12]. Metabolic engineering strategies to reduce endogenous hexose transporter affinity for glucose or to raise the affinity for xylose could be the possible ways to improve ethanol production during mixed sugar fermentations.

In *O. polymorpha* wild-type strain, Hxt1 was identified as the functional hexose transporter with high similarity to low-affinity *S. cerevisiae* Hxt1 and Hxt3 transporters. Expression of *O. polymorpha HXT1* gene was able to functionally complement growth deficiency of hexose transporter-less mutant of *S. cerevisiae*, unable to grow on hexoses [13]. However, it was reported for low-affinity glucose/xylose transporters in *S. cerevisiae* that they are induced at high glucose concentrations following the rapid degradation in the absence of glucose [10].

In *S. cerevisiae*, Hxt7 seems to be the main transporter responsible for xylose uptake when xylose is the only carbon source available or during co-fermentation when glucose reaches low levels. *GAL2* transcription is repressed in the presence of glucose, but when constitutively expressed, Gal2 and Hxt7 exhibited the highest xylose uptake rates among the endogenous hexose transporters [9, 11, 12].

To our knowledge, till now only *S. cerevisiae* was used to engineer sugar transporters to achieve more efficient xylose utilization. In this study, we compared xylose uptake and ethanol production during high-temperature alcoholic fermentation in the native xylose-utilizing *O. polymorpha* strains overexpressing engineered homologous Hxt1 transporter or heterologous Hxt7 or Gal2 transporters from *S. cerevisiae*. Engineering *O. polymorpha* Hxt1 transporter by substitution of asparagine to alanine residue at position 358 along with substitution of N-terminal lysine residues predicted to be the target for ubiquitination for arginine residues had the highest positive impact on simultaneous utilization of xylose and glucose during co-fermentation of these sugars.

## Results

### Expression of the modified versions of Hxt1 in *O. polymorpha hxt1Δ* mutant

Hxt1 was identified in *O. polymorpha* wild-type strain as the functional hexose transporter. The amino acid sequence of *O. polymorpha* Hxt1 has a high homology similarity to *S.* *cerevisiae* Hxt3 (59% identity, 75% similarity), Hxt6 and Hxt7 (59% identity, 73% similarity), Hxt1 (57% identity, 74% similarity) (Additional file 1: Figure S1). Overexpression of *O. polymorpha HXT1* gene in the genome of hexose transporter-less mutant of *S. cerevisiae* functionally complements growth deficiency of this strain on glucose [13, 14].

In this study, several modified versions of Hxt1 were constructed and the effects of these modifications on xylose and glucose co-consumption during fermentation were analyzed after introduction into genome of *hxt1Δ* mutant of *O. polymorpha*. The first version of Hxt1, named Hxt1_N358A, was engineered by substitution of asparagine to alanine at position 358 similarly to substitution N367A of *S. cerevisiae* chimeric Hxt36 transporter, which was identified as efficient for construction of glucose-insensitive xylose transporter [8].

The second version of Hxt1, named Hxt1_K, was constructed by replacement of N-terminal lysine residues predicted to be targets for ubiquitination by arginine residues. The prevention of ubiquitination is a way to reduce catabolite degradation and increase the retention of hexose transporters also in the absence of glucose in the medium [10].

The third version of Hxt1, named Hxt1_N358A_K, combined both above mentioned modifications (Additional file 1: Figure S2, Additional file 1: Figure S3).

**Fig. 1:**
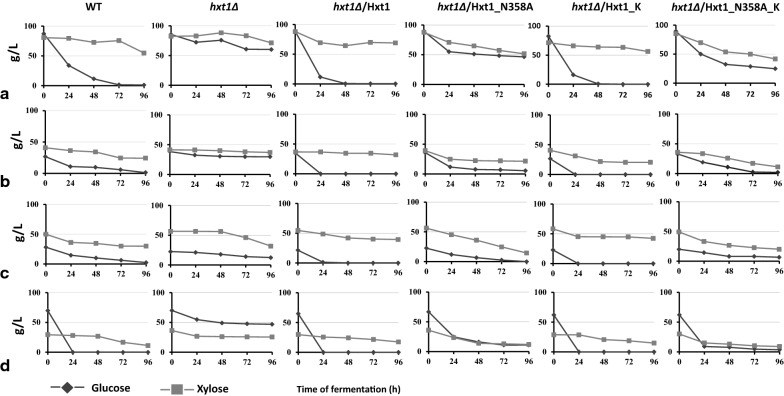
Glucose and xylose consumption by the wild type strain (WT), *hxt1Δ* mutant and obtained strains *hxt1Δ*/Hxt1, *hxt1Δ*/Hxt1_N358A, *hxt1Δ*/Hxt1_K, *hxt1Δ*/Hxt1_N358A_K during alcoholic fermentation at 45 C in the media with different xylose to glucose ratio **a** 10% xyl/10% glu **b** 5% xyl/5% glu **c** 7% xyl/3% glu **d** 7% glu/3% xyl

**Fig. 2:**
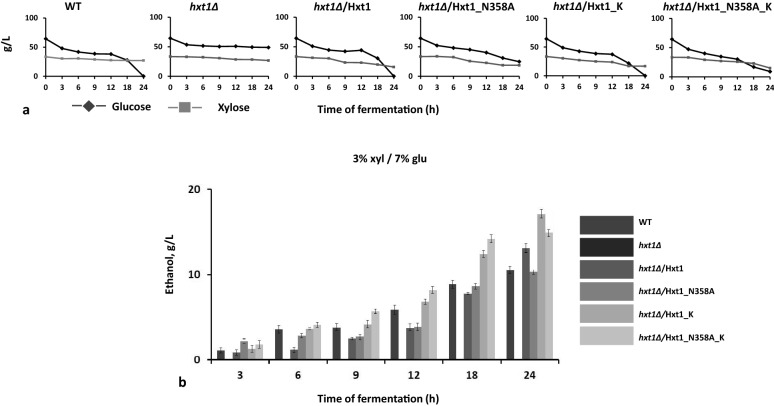
Glucose and xylose consumption (**a**) and ethanol production (**b**) by the wild-type strain (WT), *hxt1Δ* mutant and obtained strains *hxt1Δ*/Hxt1, *hxt1Δ/*Hxt1_N358A, *hxt1Δ/*Hxt1_K, *hxt1Δ/*Hxt1_N358A_K during alcoholic fermentation within 24 h at 45 °C in the medium with 7% glucose and 3% xylose

**Fig. 3:**
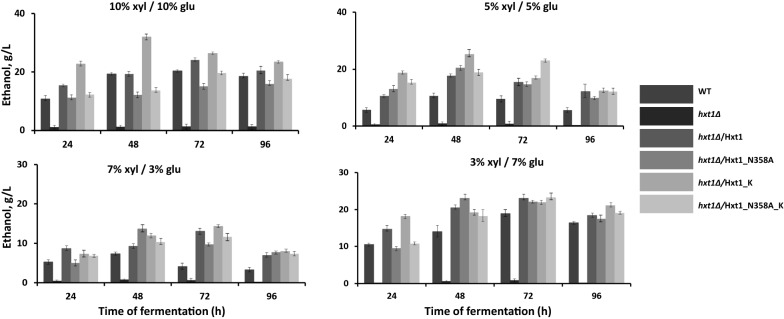
Ethanol production by the wild-type strain (WT), *hxt1Δ* mutant and obtained strains *hxt1Δ*/Hxt1, *hxt1Δ/*Hxt1_N358A, *hxt1Δ/*Hxt1_K, *hxt1Δ/*Hxt1_N358A_K during alcoholic fermentation at 45 °C in the media with different xylose to glucose ratio (xyl/glu)

The native or modified versions of Hxt1 were overexpressed in *hxt1Δ* mutant and the efficiency of xylose and glucose co-utilization during high-temperature alcoholic fermentation was compared in the obtained recombinant strains. The kinetics of consumption of both sugars during mixed sugar fermentation with different ratio of xylose and glucose was studied (Fig. 1). In the wild-type strain, a sequential consumption of glucose and xylose was observed. Glucose was totally consumed from all tested media faster than xylose, but only 40%–60% of xylose was consumed during fermentation. The *hxt1Δ* mutant was practically unable to utilize both glucose and xylose, as a result, no ethanol was produced during mutant cells incubation with these sugars (Figs. 1 and 2). The overexpression of *HXT1* wild-type allele in *hxt1Δ* mutant restored the ability to utilize sugars in a manner similar to that in the wild-type strain. The *hxt1Δ*/Hxt1_K strain also showed the bi-phase sugar consumption and at the end of fermentation, a significant amount of xylose remained unutilized. In contrast, the *hxt1Δ*/Hxt1_N358A and *hxt1Δ*/Hxt1_N358A_K variants both showed co-consumption of glucose and xylose. However, at the end of fermentation, xylose still was not completely consumed (Fig. 1). We also compared the dynamics of glucose and xylose consumption within 24 h of alcoholic fermentation in the medium with 7% glucose and 3% xylose. The glucose to xylose ratio 7% to 3% is the closest to the ratio between these sugars in lignocellulosic hydrolysates [11, 15]. The *hxt1Δ*/Hxt1_N358A_K was able to consume 87% of glucose and 50% of xylose within 24 h of fermentation, while the *hxt1Δ*/Hxt1_N358A consumed only 65% of glucose and 38% of xylose (Fig. 2a). Despite the difference in the dynamics of sugars utilization between transformants with *HXT1* modifications, all of them were characterized by slightly improved ethanol production as compared to the wild-type strain (Fig. 3). In the medium with such concentration of sugars, the overexpression of native or modified versions of *HXT1* in *hxt1Δ* mutant resulted in 30% increased ethanol production at 48 and 72 h of alcoholic fermentation as compared to the wild-type strain (Fig. 3). Within 24 h of fermentation the highest amount of ethanol was accumulated by *hxt1Δ*/Hxt1_K and *hxt1Δ*/Hxt1_N358A_K strains, however the *hxt1Δ*/Hxt1_K consumed all glucose in contrast to the *hxt1Δ*/Hxt1_N358A_K which co-utilized both sugars (Fig. 2b). Therefore, we have focus on Hxt1_N358A_K variant in our further study.

**Fig. 4:**
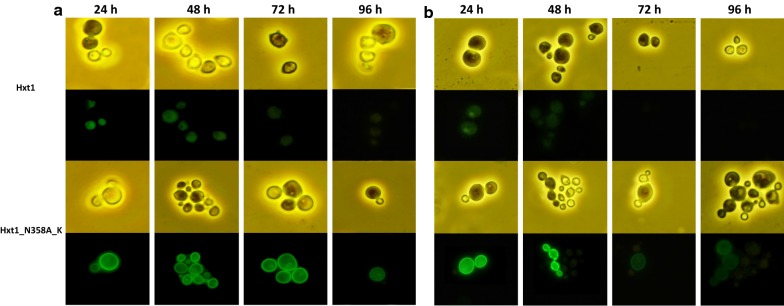
Membrane localization of Hxt1 and Hxt1_ N358A_K fused to GFP when grown on minimal medium with 2% xylose (**a**) or 2% glucose (**b**) in a 24 to 96 h time range

To determine if the improved kinetics of xylose consumption is a result of a longer retention of the mutated Hxt1 transporter in the cytoplasmic membrane, Hxt1 and Hxt1_N358A_K versions were fused C-terminally to the fluorescent reporter GFP. These fusion proteins were expressed in the *hxt1Δ* mutant. The cellular localization of the Hxt1-GFP fusion protein was assessed by fluorescence microscopy at 24 h, 48 h, 72 h and 96 h (Fig. 4). The Hxt1_N358A_K-GFP fusion localized to the plasma membrane even after 96 h of cultivation in the minimal medium with xylose. In contrast, the native Hxt1 seems to be rapidly degraded from the plasma membrane when cells were grown on xylose (Fig. 4a). In the medium with glucose the Hxt1 protein degraded more rapidly in both strains as compared to xylose containing medium, however the mutated version retained in the membrane for a longer time (Fig. 4b). Thus, the mutagenesis of the three lysine residues in Hxt1 had a notable effect on membrane retention of Hxt1.

### Expression of the engineered Hxt1, Gal2 and Hxt7 in *O. polymopha*

In our previous study, the advanced *O. polymorpha* ethanol producer from xylose was isolated by a combination of methods of metabolic engineering and classical selection. Despite increased ethanol production from xylose, the consumption of this pentose by the recombinant strain BEP *cat8Δ* was insufficient and strongly inhibited by glucose [2]. The BEP *cat8Δ* was used as a recipient to evaluate and compare the impact of the introduction of the overexpressed engineered endogenous Hxt1 or heterologous Hxt7 and Gal2 transporters on sugar consumption and alcoholic fermentation performance. To achieve this goal, the Hxt1_N358A_K was overexpressed into the genome of BEP *cat8Δ* under control of the strong constitutive *GAP* promoter. The modified versions of heterologous Hxt7 and Gal2 transporters from *S. cerevisiae* were constructed and also overexpressed in BEP *cat8Δ* strain. The Gal2 was modified by the substitution of asparagine to serine at position 376 and the Hxt7 by the substitution of the corresponding asparagine to phenylalanine at position 370 [9] (Additional file 1: Figure S3). The expression of genes coding modified transporters was confirmed using qRT-PCR. The expression of *HXT1* in BEP *cat8Δ*/Hxt1_N358A_K strain was increased by 8 times as compared to the parental BEP *cat8Δ* strain and *GAL2* in BEP *cat8Δ*/Gal2_N376S. The expression of *HXT7* in BEP *cat8Δ*/Hxt7_N370F and *GAL2* in BEP *cat8Δ*/Gal2_N376S strains was increased by 6 times.

**Fig. 5:**
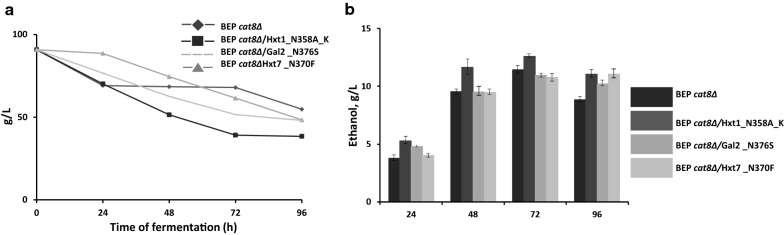
Xylose consumption (**a**) and ethanol production (**b**) by the parental BEP/cat8 strain and obtained strains Hxt1_N358A_K, Gal2_N376S, Hxt7_N370F during alcoholic fermentation at 45 °C in the medium with 9% xylose

The ethanol production and the kinetics of glucose and xylose consumption during high-temperature alcoholic fermentation were compared in recombinant strains carrying the modified Hxt1, Hxt7, and Gal2 transporters. The strain with modified Hxt1 was characterized by the most rapid xylose consumption among all tested transformants during xylose fermentation. After 96 h of fermentation, the BEP *cat8Δ*/Hxt1_N358A_K strain was able to consume 49% more xylose than the parental strain, when the BEP *cat8Δ*/Gal2_N376S strain consumed only 20% more xylose. The BEP *cat8Δ*/Hxt7_N370F strain within 48 h of fermentation consumed xylose even slower than that of the wild-type strain, but after 96 h of fermentation, the uptake of xylose was improved by 20%. The BEP *cat8Δ*/Hxt1_N358A_K strain was also characterized by the a slightly increased ethanol production during xylose fermentation, reaching 20% more ethanol at 48 h (Fig. 5).

**Fig. 6:**
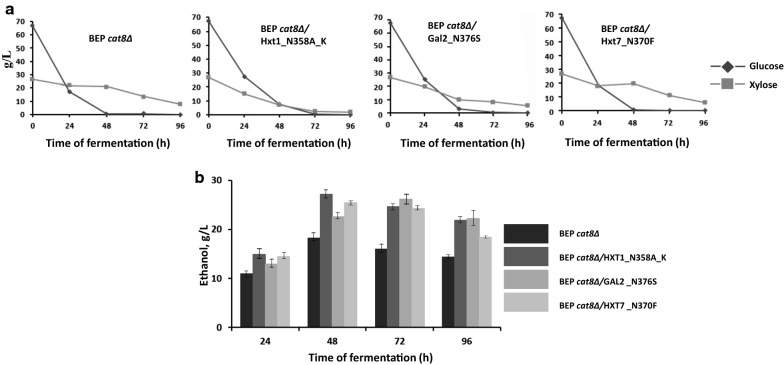
Glucose and xylose consumption (**a**) and ethanol production (**b**) by the parental BEP/cat8 strain and obtained strains Hxt1_N358A_K, Gal2_N376S, Hxt7_N370F during alcoholic fermentation at 45 °C in the medium with 7% glucose and 3% xylose

The uptake of glucose and xylose by strains with modified Hxt1, Hxt7 and Gal2 was also compared during fermentation in the medium with 7% of glucose and 3% of xylose. The parental BEP *cat8Δ* strain was able to consume all glucose and only 30% of xylose within 48 h. The uptake of both sugars by the strain with modified Hxt7 was the same as in the parental strain. At the same time, the strains, carrying modified Hxt1 and Gal2, consumed 73% and 62% more of xylose, respectively. However, ethanol production was increased during fermentation in all tested strains as compared to the parental strain (Fig. 6, Table 1). The ethanol yield and ethanol productivity of strains with modified Hxt1, Hxt7 and Gal2 was also higher as compared to the parental strain BEP *cat8Δ*. At the fermentation temperature of 45 °C BEP *cat8Δ*/Hxt1_N358A_K strain gave the highest ethanol yield 0.35 g ethanol/g sugar that is 30% more as compared to the BEP *cat8Δ* (Table 1).

**Table 1:** Main parameters of glucose and xylose co-fermentation (7:3) on 48 h at 45 °C by the *O. polymorpha* strains tested

Strain	Ethanol (g/L)	Ethanol yield (g/g consumed substrate)	Rate of ethanol synthesis (g/g biomass/h)	Productivity of ethanol synthesis (g/L/h)
BEP *cat8Δ*	18.23 ± 0.096	0.256 ± 0.009	0.165 ± 0.004	0.380 ± 0.016
BEP *cat8Δ*/Hxt1_N358A_K	27.56 ± 0.127	0.347 ± 0.014	0.212 ± 0.008	0.574 ± 0.029
BEP *cat8Δ*/Gal2 _N376S	22.59 ± 0.089	0.310 ± 0.011	0.188 ± 0.004	0.471 ± 0.019
BEP *cat8Δ/*Hxt7 _N370F	25.44 ± 0.065	0.319 ± 0.014	0.203 ± 0.007	0.530 ± 0.024

**Fig. 7:**
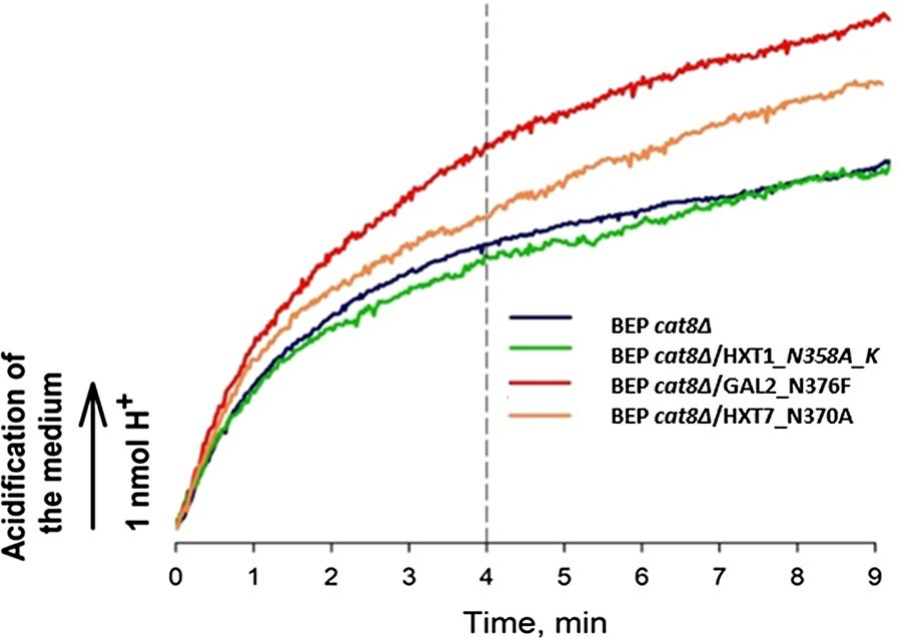
Effect of xylose on the acidification of the incubation medium by *O. polymorpha* cells taken after 24 h fermentation of xylose without starvation with 2-deoxyglucose. The incubation medium contained 5 mM sodium phosphate in solution of 95 mM NaCl, pH 7. The experiments were performed at 45 °C. Yeast cells were added at 0 min to a concentration of 6 x 10^7^cells/ml. The acidification rate was calculated for the first 4 min after glucose or xylose addition. The results shown are representative of three independent experiments

The metabolic activity of the studied cells was assayed following the acidification of the incubation medium in response to the additions of carbon substrates, xylose or glucose. We analyzed recombinant strains carrying modified Hxt1, Gal2, and Hxt7 transporters comparing them to the parental BEP *cat8Δ* cells (Fig. 7). The cells were grown in media with a mixture of glucose and xylose or xylose alone. Before the experiments, cells were starved without any carbon source. We measured the capability of the starved cells to acidify the extracellular medium upon additions of d-glucose or d-xylose. Table 2 summarizes results of the measurements.

**Table 2:** Acidification of the incubation medium after addition of substrates (nmol H^+^/min)

Conditions	Strains			
	BEP *cat8Δ*	BEP *cat8Δ*/Hxt1_N358A_K	BEP *cat8Δ*/Gal2_ N376S	BEP *cat8Δ*/Hxt7_N370F
Cells grown with glucose and xylose mixture + glucose	0.92 ± 0.06	1.0 ± 0.13	1.10 ± 0.14	0.82 ± 0.09
Cells grown with glucose and xylose mixture + xylose	0.60 ± 0.13	0.75 ± 0.09	0.63 ± 0.09	0.64 ± 0.06
Cells grown with xylose + xylose	0.50 ± 0.07	0.66 ± 0.03	0.48 ± 0.09	0.54 ± 0.12

Cells grown in the mixtures of xylose and glucose, strongly acidified the external medium after the addition of glucose. Cells grown at the same conditions, but after xylose addition, showed lower rates of acidification, similarly to the cells grown in the medium with xylose. After the addition of xylose, the most efficient acidification was performed by strain expressing Hxt1 as compared to parental BEP *cat8Δ* (Table 2).

## Discussion

Xylose is the second most abundant sugar of the lignocellulose after glucose, therefore efficient conversion of xylose by microorganisms is a significant prerequisite for the development of economically feasible technology for the production of second generation ethanol. The wild-type strains of the methylotrophic thermotolerant yeast *O. polymorpha* grow well on xylose and ferment it under semianaerobic conditions, but the amounts of accumulated ethanol are very low. The reasons of this phenomenon are not known, however, as methods of molecular genetics are well developed for this organism and genome sequence is publicly available, *O. polymorpha* is considered as a promising model organism for construction of the efficient thermotolerant ethanol producer.

A combination of metabolic engineering and classical selection approaches was successfully used for improvement of parameters of xylose alcoholic fermentation in *O. polymorpha* [2, 3, 16]. However, even recombinant strains with improved ethanol production up to 25–30 times were characterized by incomplete xylose utilization during fermentation. Therefore, the inefficient transport of xylose is considered to be one of the possible rate-limiting steps in xylose conversion to ethanol.

The uptake and consumption of xylose are insufficient and strongly inhibited by glucose due to the lack of xylose-specific transporters in the most of studied microorganisms. Xylose most often is transported by non-specific hexose transporters with poor affinity for xylose [4]. There have been many recent reports of improved transport and utilization of xylose in *S. cerevisiae* by expression of the heterologous transporters from native xylose-utilizing species [16–18], as well as evolutionary or protein engineering of endogenous hexose transporters for increase of the affinity for xylose and decrease for glucose [8–10]. It was found that all of the mutated transporters had a mutation at either asparagine 376/370 or threonine 219/213 and it is likely that these alterations sterically prevent d-glucose but not d-xylose from entering the sugar-binding pocket [9, 11]. As hexose transporter sequences are conserved, these studies could be applied to predict rational designs for rewiring new xylose transporters. In *O. polymorpha* until now only Hxt1 was identified as the functional hexose transporter with high similarity to low-affinity Hxt1 and Hxt3 transporters of *S. cerevisiae* [13].

Here, we studied the role of *O. polymorpha* engineered Hxt1 transporter in xylose utilization during high-temperature alcoholic fermentation of xylose or mixture of xylose and glucose. Moreover, several engineering approaches formerly used for low-affinity glucose transporters in *S. cerevisiae* were applied for modification of *O. polymorpha* Hxt1 transporter. Engineering *O. polymorpha* Hxt1 by substitution of asparagine to alanine residue at position 358 along with substitution of N-terminal lysine residues predicted to be the target for ubiquitination for arginine residues resulted in simultaneous utilization of xylose and glucose during co-fermentation of these sugars after overexpression into genome of *hxt1Δ* mutant and advanced ethanol producer BEP *cat8Δ*. The *hxt1Δ* mutant was practically unable to utilize both glucose and xylose during co-fermentation, however was able to grow normally on 2% of glucose or xylose during cultivation under aerobic condition. During co-fermentation of 7% of glucose and 3% of xylose both sugars were almost completely utilized by strain BEP *cat8Δ*/Hxt1_N358A_K expressing the modified version of Hxt1 in contrast to the parental BEP *cat8Δ*. However, a significant amount of xylose remained unutilized during fermentation of xylose alone by either BEP *cat8Δ* or BEP *cat8Δ*/Hxt1_N358A_K strains. These results suggest that Hxt1 is not crucial for xylose utilization but is involved in xylose transport in *O. polymorpha*. Therefore, the screening for other putative endogenous xylose transporters and other relevant genes seems to be a promising way to construct an efficient xylose fermenting strains.

We also compared xylose uptake and ethanol production during high-temperature alcoholic fermentation by *O. polymorpha* strains overexpressing engineered endogenous Hxt1 transporter with *O. polymorpha* strains overexpressing engineered heterologous Hxt7 or Gal2 transporters from *S. cerevisiae*. The expression of the modified Hxt1 resulted in simultaneous utilization of both glucose and xylose during fermentation in the constructed *O. polymorpha* recombinant strains. The overexpression of heterologous Gal2 had a less pronounced effect on sugars uptake rate, while overexpression of Hxt7 revealed the least impact on sugars consumption.

The best reported industrial *S. cerevisiae* yeast strain was able to consume both glucose and xylose with an ethanol yield of 0.46 at 32 °C, however, the fermentation rate was significantly reduced at 39 °C [19, 20]. The thermotolerant isolates of *Pichia kudriavzevii* and *Candida tropicalis* fermented a mixture of glucose and xylose (6:4) with ethanol yield 0.33 and 0.36 g ethanol/g sugar, respectively, at 42 °C [21]. *Spathaspora passalidarum* that is capable of co-fermenting xylose and cellobiose in the presence of glucose under oxygen-limiting conditions was able to produce 0.33 g/L ethanol/g sugar at 40 °C during mixed sugar fermentation [22]. In this study the *O. polymorpha* strains overexpressing engineered endogenous Hxt1 transporter was obtained able to produce 0.35 g/L ethanol/g sugar at 45 °C during co-fermentation of glucose and xylose.

The overall metabolic activity of the studied cells in response to the additions of xylose or glucose was monitored by an electrochemical pH registration system. The cells were grown in the mixture of glucose and xylose or xylose alone. The medium acidification rate of the starved cells could be used as an indicator of the intensity of carbon catabolism upon the addition of d-glucose or D-xylose to suspensions [23]. Our data suggest that overexpression of the engineered *HXT1* in *O. polymorpha* cells resulted in the highest rate of extracellular acidification after addition of xylose as compared to the heterologous engineered transporters Gal2 and Hxt7.

## Conclusions

In the current study, the impact of the engineered *O. polymorpha* Hxt1 and *S. cerevisiae* Gal2 and Hxt7 transporters on xylose and glucose utilization and ethanol production in *O. polymorpha* were compared under conditions of high-temperature alcoholic fermentation. The expression of endogenous engineered Hxt1 in *O. polymorpha* had the most pronounced effect on simultaneous sugars consumption and ethanol production during co-fermentation of glucose and xylose as compared to engineered heterologous Gal2 or Hxt7 of *S. cerevisiae*.

## Methods

### Strains, media, and culture conditions

*Ogataea polymorpha* cells were grown on YPD (10 g/L yeast extract, 10 g/L peptone, 20 g/L glucose) or mineral (6.7 g/L YNB without amino acids, 20 g/L of glucose or xylose) media at 37 °C. For the NCYC495 *leu1*-*1* strain, leucine (40 mg/L) was added to the medium. For selection of yeast transformants on YPD, 0.1 g/L of nourseothricin or 0.2 g/L of zeocin were added. Alcoholic fermentation of *O. polymorpha* cells was tested at 45 °C as described previously [2] in the medium with 10% xylose or mixtures of glucose and xylose with ratios 7%: 3%, 5%: 5%, or 3%: 7%. The fermentation experiments were performed in at least triplicate to ensure the reproducibility of results. Bars in the figures indicate ranges of the standard deviation.

For electrochemical measurements, cells were taken after 24 h cultivation under fermentation conditions with 10% xylose or mixture of glucose and xylose (7%: 3%). The cells were pelleted, washed with 5 mM sodium phosphate buffer in 95 mM NaCl, pH 7.0 and starved with or without 2-deoxyglucose for 1 h.

*E.* *coli DH5α* strain (Φ80d*lacZ*ΔM15, *recA*1, *endA*1, *gyrA*96, *thi*-1, *hsdR*17(rK^−^, mK^+^), *supE*44, *relA*1, *deoR*, Δ(*lacZYA*-*argF*)U169) was used as a host for plasmid propagation. *DH5α* cells were grown at 37 °C in LB medium as described previously [24]. Transformed *E. coli* cells were maintained on a medium containing 100 mg/L of ampicillin.

### Construction *O. polymorpha* strains with engineered endogenous Hxt1 or *S.* *cerevisiae* Hxt7 and Gal2 transporters

*Ogataea polymorpha* genome database (https://mycocosm.jgi.doe.gov/Hanpo2/ Hanpo2.info.html) was used for retrieval of *HXT1* gene sequence. The open reading frame of *HXT1* gene along with its own gene terminator was amplified from *O. polymorpha* NCYC495 genomic DNA using primers OK161/OK162 (Additional file 2: Table S1). The native promoter of this gene was substituted by a strong constitutive promoter of glyceraldehyde-3-phosphate dehydrogenase (*GAP1*).

The promoter of *GAP1* gene was amplified from *O. polymorpha*NCYC495 genomic DNA using primers OK159/OK160. The resulting fragments were fused by overlap PCR using primers OK159/OK162. The amplified fragment (2.6 kb) was XbaI/SphI digested and cloned into the corresponding sites of the pUC57/zeo vector, carrying selective marker gene conferring resistance to zeocin. The constructed plasmid was named pUC19/zeo/HXT1 (Additional file 1: Figure S3) and used as a template for the construction of modified versions of Hxt1.

One version of Hxt1 was engineered by substitution of asparagine to alanine at position 358 to increase the specific xylose uptake rate and decrease affinity to glucose. Primers OK163/OK164 and OK165/OK162 were used for amplification of N- and C-fragments of *HXT1* gene. The resulting fragments were fused by overlap PCR using primers OK163/OK162. The amplified fragment (1.6 kb) was NotI/SphI digested and cloned into the corresponding sites of the pUC19/zeo/HXT1 vector instead of the native version of *HXT1*gene. The constructed plasmid was named pUC19/zeo/HXT1_N358A (Additional file 1: Figure S3).

The predicted targets of ubiquitination were identified in the sequence of *HXT1* gene using UbPred program software http://www.ubpred.org. The N-terminally located lysine residues at positions 8, 9 and 30 identified as potentially involved in ubiquitination were replaced for by arginine residues. Primers OK159/OK167 and OK166/OK162 were used for amplification of *GAP1* promoter with N-fragment and C-fragments of *HXT1* gene, respectively. The resulting fragments were fused by overlap PCR using primers OK159/OK162. The amplified fragment (2.6 kb) was XbaI/SphI digested and cloned into the corresponding sites of the pUC19/zeo vector. The constructed plasmid was named pUC19/zeo/HXT1_K (Additional file 2: Figure S3).

The modified version of *HXT1* with all mentioned modifications was obtained by PCR from the plasmid pUC19/zeo/HXT1_K using primers OК163/OК164 and OК165/OК162. The resulting fragments were fused by overlap PCR using primers OK163/OK162. The amplified fragment (1.6 kb) was NotI/SphI digested and cloned into the corresponding sites of the pUC19/zeo/HXT1 vector instead of the native version of *HXT1* gene. The constructed plasmid was named pUC19/zeo/HXT1_N358A_K (Additional file 1: Figure S3).

Primers OK161/OK216 were used for amplification of the corresponding fragment (1.6 kb) from the plasmid pUC19/zeo/HXT1_N358A_K. The resulting fragment was NotI-digested and cloned into the corresponding site of the pUC19_prGAP_NTC [25]. The constructed plasmid was named pNTC_HXT1_N358A_K (Additional file 1: Figure S3).

The *S. cerevisiae* genome database (https://www.yeastgenome.org/) was used for retrieval of *HXT7* and *GAL2* gene sequences. The modified version of Hxt7 was constructed by replacement of asparagine residue at position 370 for serine. Primers OK203/OK204 and OK205/OK217 were used for amplification of N- and C-fragments of *HXT7* gene from a genomic DNA of *S. cerevisiae* BY4742. The resulting fragments were fused by overlap PCR using primers OK203/OK217. The amplified fragment (1.7 kb) was XbaI digested and cloned into the corresponding site of the pUC19_prGAP_NTC. The constructed plasmid was named pNTC_HXT7_N370A (Additional file 1: Figure S3).

Primers OK207/OK208 and OK209/OK218 were used for amplification of N- and C-fragments of *GAL2* gene from a genomic DNA of *S. cerevisiae* BY4742. The resulting fragments were fused by overlap PCR using primers OK207/OK218. The amplified fragment (1.7 kb) was XbaI digested and cloned into the corresponding site of the pUC19_prGAP_NTC. The constructed plasmid was named pNTC_GAL2_N376F (Additional file 1: Figure S3).

### Visualization of membrane localization of Hxt1 in *O. polymorpha* by fluorescence microscopy

The DNA fragment harboring the gene coding for the green fluorescent protein (GFP) was amplified using primers Ko819 and Ko820 from the plasmid pGFP-SLK [3]. The backbone plasmids containing *HXT1* or *HXT1*_N358A_K was amplified with the primers Ko821/Ko822 from the plasmids pUC19/zeo/HXT1 or pUC19_prGAP_NTC_HXT1_ N358A_K. Two PCR fragments were then Gibson assembled to generate the plasmids pUC19/zeo/HXT1_GFP or pUC19_prGAP_NTC_HXT1_ N358A_K_GFP. These plasmids were introduced into the genome of *O. polymorpha hxt1Δ* mutant. Transformants were selected on solid YPD medium supplemented with 0.2 g/L of zeocin after 3 days of incubation. Selected transformants were stabilized by alternating cultivation in non-selective and selective media and examined by diagnostic PCR using primers, OK161/Ko820. The resulting strains were grown at 37 °C in YNB medium with xylose during 96 h followed by microscopy analysis. Images were captured on a fluorescence microscope Axio Imager A1 (Carl Zeiss MicroImaging, Jena, Germany) coupled to a monochrome digital camera Axio Cam MRm (Carl Zeiss MicroImaging) and processed using the AxioVision 4.5 (Carl Zeiss MicroImaging) and Adobe Photoshop CC software (Adobe Systems, Mountain View, CA).

### Quantitative real–time PCR (qRT–PCR)

Expression of the *HXT1*, *HXT7* and *GAL2* genes was analyzed by real-time PCR. The qRT-PCR was performed by 7500 Fast RealTime PCR System (Applied Biosystems, USA) with SG OneStep qRT-PCR kit (EURx Ltd., Gdansk, Poland) using gene-specific pairs of primers, RNA as a template, and ROX reference passive dye according to the manufacturer’s instructions as described previously [2]. The pairs of primers used for qRT-PCR are listed in Additional file 1: Table S1. Sequences of the tested genes were taken from *O. polymorpha* genome database.

### Analyses

The optical density (OD) of cell suspensions for biomass determination was measured using a « Helios-λ » spectrophotometer at λ 590 and λ 663 nm for *O. polymorpha* transformation [26]. Concentrations of xylose and ethanol from fermentation in the medium broth were analyzed by HPLC (PerkinElmer, Series 2000, USA) with an Aminex HPX-87H ion-exchange column (Bio-Rad, Hercules, USA). A mobile phase of 4 mM H2SO4 was used at a flow rate 0.6 ml/min and the column temperature was 30 °C. Alternatively, concentrations of ethanol in the medium were determined using alcohol oxidase/peroxidase-based enzymatic kit “Alcotest” [27]. Experiments were performed at least twice.

### Measurements of the extracellular acidification

The extracellular acidification was determined by pH electrode (Hanna Instruments HI1131B), measuring pH from 0 to 13 at −5 to 100 °C. The software NT-MDT Nova 850 and the computer program LabChart were used to monitor changes in the concentrations of hydrogen ions in the test solutions. Measurements were performed in 9 mL of 5 mM sodium phosphate-75 mM NaCl buffer, pH 7. At the beginning of every experiment, the signal was allowed to settle before the cells were added into the test solution. Once the signal has settled, 2% d-xylose or 2% d-glucose were added. At the end of the experiment, the known concentration of NaOH was added for calibration.

## Supplementary information

**Additional file 1: Figure S1.** Alignment of amino acid sequences of *O. polymorpha* Hxt1 and S. cerevisiae Hxt1, Hxt3, Hxt6, Hxt7 transporters. **Figure S2.** Sequence of *O. polymorpha* Hxt1 transporter. The lysine residues substituted for arginine are shaded grey. The position of the asparagine residue that was mutated to an alanine to obtain Hxt1-N358A mutant is underlined. **Figure S3.** Linear schemes of plasmids for overexpression of the modified versions of Hxt1, Gal2 and Hxt7 transporters.

**Additional file 2: Table S1.** List of primers used in this study.

## Data Availability

The materials and datasets for the current study are available from the corresponding author on reasonable request.

## Abbreviations

SSF: Simultaneous saccharification and fermentation
qRT-PCR: Quantitative reverse-transcription PCR
BEP: Best ethanol producer
