# Supplementary material for: Engineering of sugar transporters for improvement of xylose utilization during high-temperature alcoholic fermentation in Ogataea polymorpha yeast
Source: Microb Cell Fact. 2020 Apr 25;19:96. doi: 10.1186/s12934-020-01354-9 (PMC7183630; doi:10.1186/s12934-020-01354-9)
Supplement: Supplementary file 1 — Additional file 1: Figure S1. Alignment of amino acid sequences of O. polymorpha Hxt1 and S. cerevisiae Hxt1, Hxt3, Hxt6, Hxt7 transporters. Figure S2. Sequence of O. polymorpha Hxt1 transporter. The lysine residues substituted for arginine are shaded grey. The position of the asparagine residue that was mutated to an alanine to obtain Hxt1-N358A mutant is underlined. Figure S3. Linear schemes of plasmids for overexpression of the modified versions of Hxt1, Gal2 and Hxt7 transporters. [file 12934_2020_1354_MOESM1_ESM.pptx]

## Slide 1
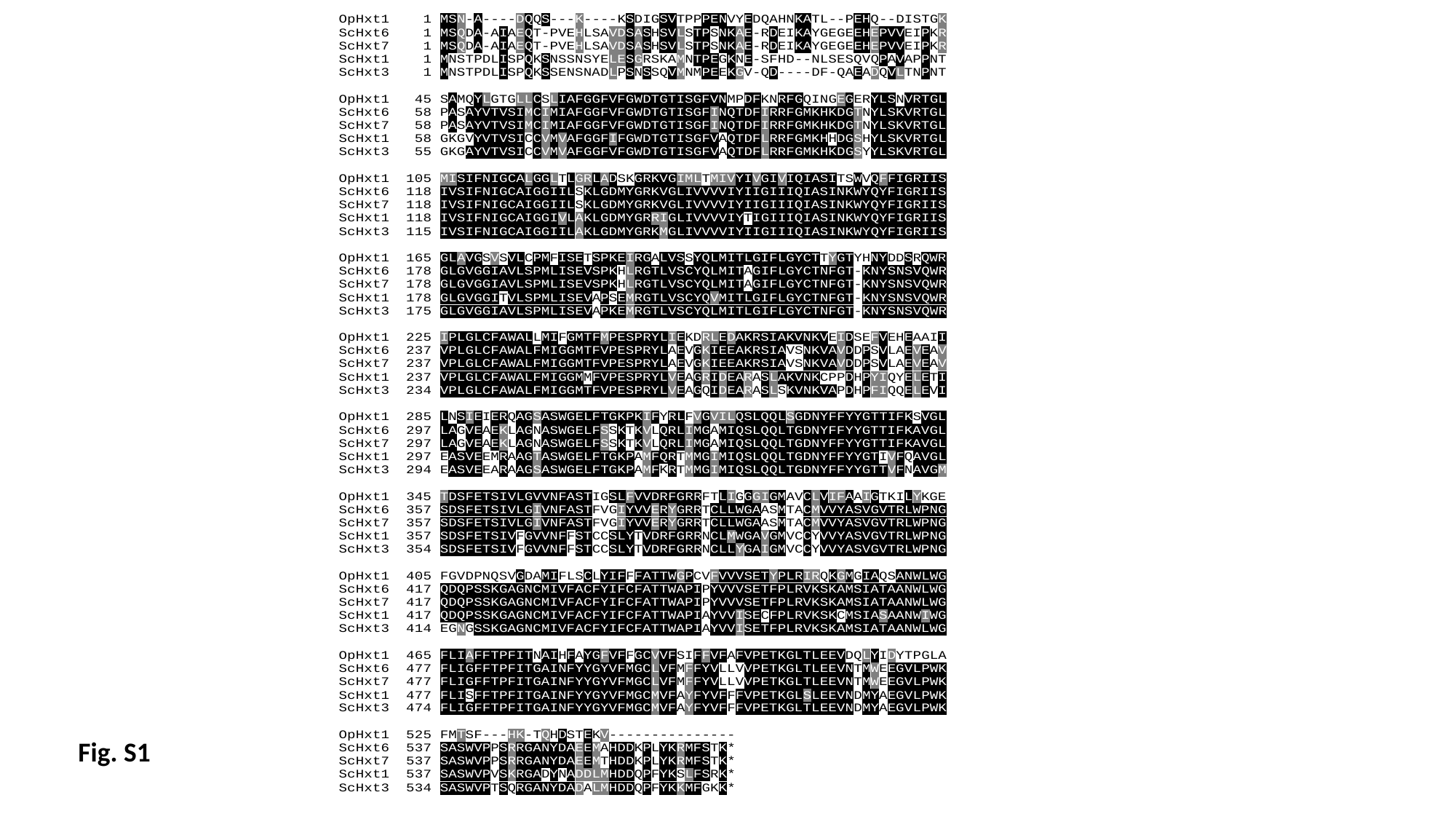

Fig. S1

## Slide 2
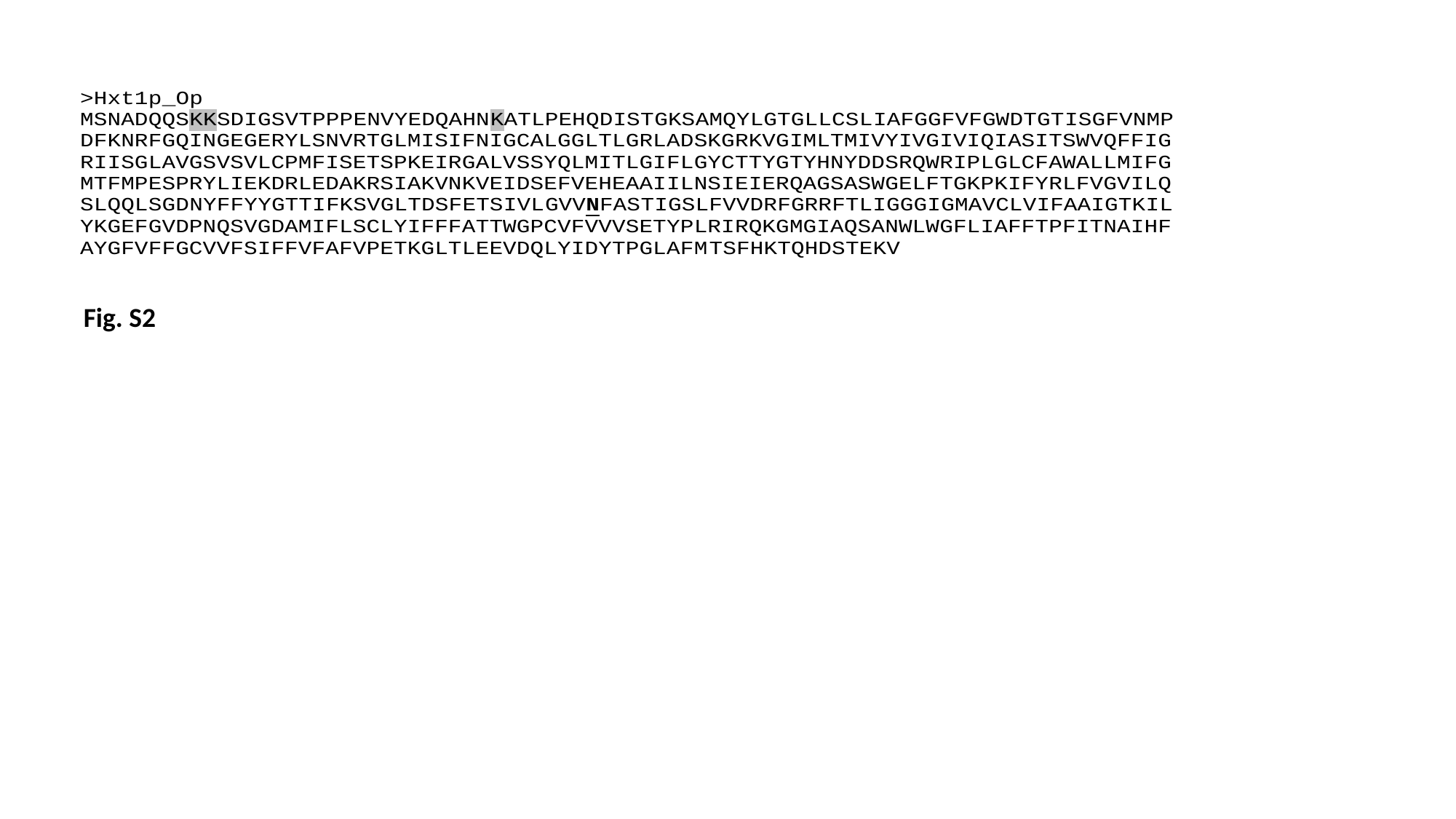

Fig. S2

## Slide 3
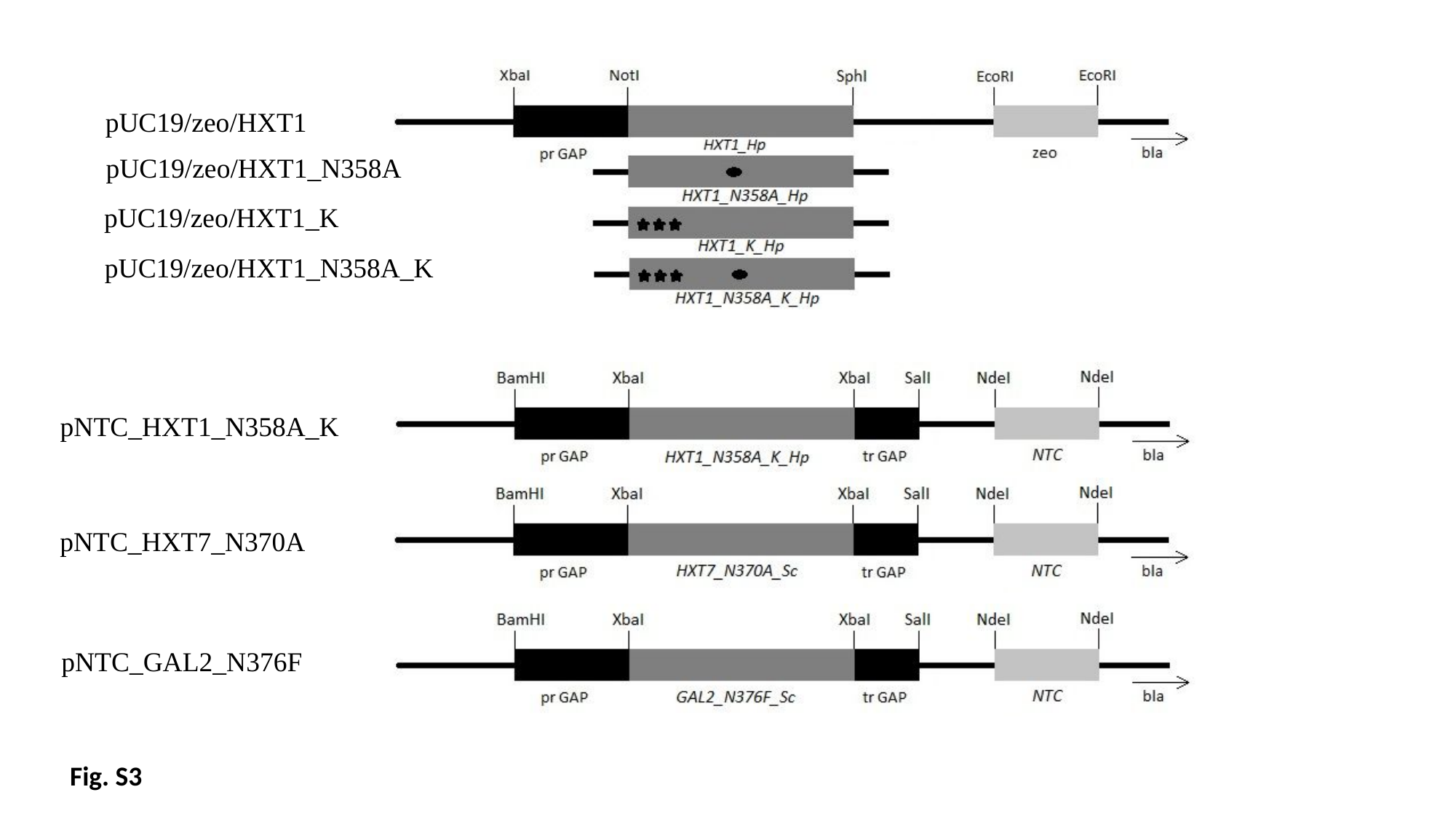

pUC19/zeo/HXT1
pUC19/zeo/HXT1_N358A
pUC19/zeo/HXT1_K
pUC19/zeo/HXT1_N358A_K
pNTC_HXT1_N358A_K
pNTC_HXT7_N370A
pNTC_GAL2_N376F
Fig. S3
