# Supplementary material for: Engineering of sugar transporters for improvement of xylose utilization during high-temperature alcoholic fermentation in Ogataea polymorpha yeast
Source: Microb Cell Fact. 2020 Apr 25;19:96. doi: 10.1186/s12934-020-01354-9 (PMC7183630; doi:10.1186/s12934-020-01354-9)
Supplement: Supplementary file 2 — Additional file 2: Table S1. List of primers used in this study. [file 12934_2020_1354_MOESM2_ESM.docx]

**Table S1** List of primers used in this study ^a^

| **Primer** | **Sequence 5’ – 3’** |
| --- | --- |
| OK159 | TGC TCTAGA TAG ACC ACA TCC GTG CAC CAG |
| OK160 | GTT GAT CCG CGT TAG ACA TGC GGC CGC TTT GTT TCT ATA TTA TCT TTG TAC TAA A |
| OK161 | TTT AGT ACA AAG ATA ATA TAG AAA CAA AGC GGC CGC ATG TCT AAC GCG GAT CAA C |
| OK162 | CAT GCATGC TTA AAA GTG GTC CGA GGA G |
| OK163 | TTT GCGGCCGC ATG TCT AAC GCG GAT CAA CAA TCC AAA AAA TC |
| OK164 | GA GGC AAA **GGC** GAC AAC ACC AAG AAC |
| OK165 | GTT CTT GGT GTT GTC **GCC** TTT GCC TC |
| OK166 | CAA CAA TCC **AGA** **AGA** TCT GAT ATT GGC TCT GTC ACG CCT CCT CCG GAG AAC GTG TAC GAG GAC CAA GCA CAC AAC **AGG** GCA AC |
| OK167 | GT TGC **CCT** GTT GTG TGC TTG GTC CTC GTA CAC GTT CTC CGG AGG AGG CGT GAC AGA GCC AAT ATC AGA **TCT TCT** GGA TTG TTG |
| OK203 | TGC TCTAGA ATG TCA CAA GAC GCT GCT ATT G |
| OK204 | TGC TCT AGA ATG TCA CAA GAC GCT GCT ATT G |
| OK205 | GTC TTG GGT ATT GTT AGC TTT GCT TCC |
| OK207 | TGC TCTAGA ATG GCA GTT GAG GAG AAC |
| OK208 | GT GGA GGC AAA GAA GAC TAC ACC |
| OK209 | GGT GTA GTC TTC TTT GCC TCC AC |
| OK216 | TTT GCGGCCGC TTA GAC CTT TTC AGT GGA GTC GTG |
| OK217 | TGC TCTAGA TTA TTT GGT GCT GAA CAT TC |
| OK218 | TGC TCTAGA TTA TTC TAG CAT GGC CTT G |

^a)^ restriction sites are underlined
